# ETHOS.REFLOW: An open-source workflow for reproducible renewable energy potential assessments

**DOI:** 10.1016/j.patter.2025.101172

**Published:** 2025-02-04

**Authors:** Tristan Pelser, Jann Michael Weinand, Patrick Kuckertz, Detlef Stolten

**Affiliations:** 1 Institute of Climate and Energy Systems, Jülich Systems Analysis, Forschungszentrum Jülich, Jülich, Germany; 2 RWTH Aachen University, Chair for Fuel Cells, Faculty of Mechanical Engineering, 52062 Aachen, Germany

**Keywords:** open-source workflow, reproducibility, transparency, wind potential assessment, technical potential, offshore wind, North Sea, floating turbines

## Abstract

Accurate renewable energy resource assessments are necessary for energy system planning to meet climate goals, yet inconsistencies in methods and data can produce significant differences in results. This paper introduces ETHOS.REFLOW, a Python-based workflow manager that ensures transparency and reproducibility in energy potential assessments. The tool enables reproducible analyses with minimal effort by automating the entire workflow, from data acquisition to reporting. We demonstrate its functionality by estimating the technical offshore wind potential of the North Sea, for fixed-foundation and mixed-technology (including floating turbines) scenarios. Two methods for turbine siting (explicit placement vs. uniform power density) and wind datasets are compared. Results show a maximum installable capacity of 768–861 GW and an annual yield of 2,961–3,047 TWh, with capacity factors between 41% and 46% and significant temporal variability. ETHOS.REFLOW offers a robust framework for reproducible energy potential studies, enabling energy system modelers to build on existing work and fostering trust in findings.

## Highlights

• Develop a workflow manager for reproducible, transparent energy potential assessments
• ETHOS.REFLOW automates the data science workflow from data acquisition to reporting
• Compare two workflows estimating North Sea’s technical offshore wind potential
• Maximum annual power yield of 2,961–3,047 TWh for North Sea’s wind resource

## The bigger picture

Global efforts to mitigate climate change involve transitioning energy systems toward renewable energy sources, including wind and solar photovoltaics (PV). Due to the variable nature of these energy sources, integration into existing energy systems is complex and requires a high degree of planning. An important input to energy system models is the “energy potential” for a given technology, referring to the maximum achievable installable capacity or power yield for the study area. Inconsistencies in methods, data, and assumptions in resource potential assessments may hinder accurate energy system planning. To address this gap, we develop ETHOS.REFLOW, a Python-based workflow manager for automating renewable energy potential analyses. We demonstrate the tool’s utility through an assessment of the North Sea’s technical wind potential. We employ REFLOW to compare two distinct workflows, illustrating how the tool can be used to ensure reproducibility and to help energy system modelers compare workflows.

## Graphical abstract

**Figure:**
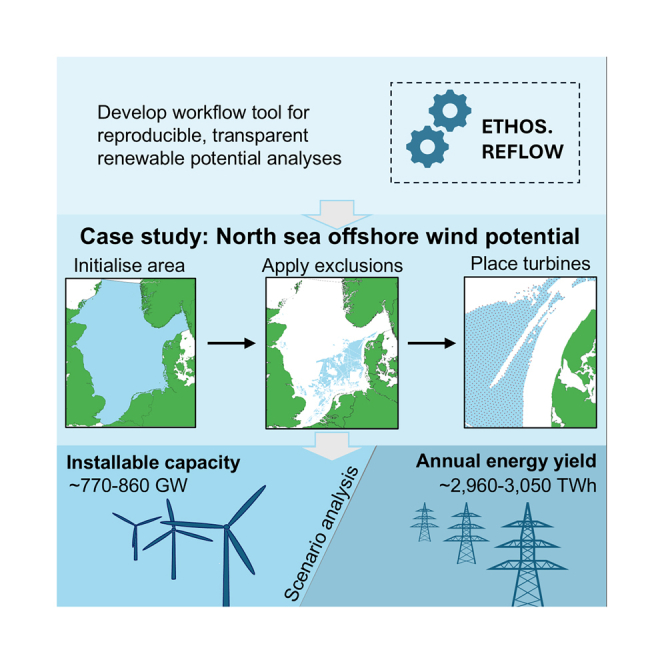

## Teaser

ETHOS.REFLOW is an open-source Python workflow manager for automating renewable energy potential assessments with the goal of improving reproducibility and transparency. In this article, the authors introduce the tool’s core utilities and then estimate the technical offshore wind potential for the North Sea comparing two methodologies and input wind data sources. They demonstrate how the tool can be used by energy system modelers to compare workflows and to illustrate where uncertainties are introduced.

## Introduction

To achieve their national climate mitigation goals, almost all developed economies have set plans for the future development of their energy systems with a high penetration of variable renewable energy sources.^1^^,^^2^^,^^3^ Accurate estimates of the resource potential for renewable technologies are crucial for modeling and planning future energy systems. However, the results of these analyses can vary greatly depending on the data employed, the methodology of the study and the research team’s assumptions relating to parameters that influence the total energy generation or feasibility of potential renewable energy projects.^4^^,^^5^ Illustrating this point, energy system modelers have previously estimated the maximum installable offshore wind capacity of the North Sea at 498,^6^ 970,^7^ 1,200,^8^ and 1,898 GW,^9^ with an annual generation of between 259^10^ and 5,615 TWh.^7^ These results imply that offshore wind power from the North Sea has the potential to supply between 6.4% and 1.4 times Europe’s total electricity generation,^11^ while the highest estimated installable capacity is 3.6 times greater than the lowest. Such large discrepancies in results could clearly have impacts on energy system planning and development policymaking.

Differences in assumptions—such as social or regulatory barriers to wind park development, technological variations (e.g., turbine availability over time and the impact of wake effects), input data selection and the models or software used—can lead to discrepancies in research outcomes. These factors are common in data-heavy fields, where the “reproducibility crisis” presents a unique challenge.^12^^,^^13^ For instance, in many analyses, replicating or building on results to account for different parameters is unfeasible due to limited data accessibility, lack of shared code, or insufficient methodological documentation. This issue is especially prominent when the research makes use of complex models that have steep learning curves and often pose significant challenges for new users. As an example, a recent review on 195 large-scale, regional wind potential assessments^4^ found that only 10% of studies provided open access to the input data, while 4% provided downloadable code. In addition, of the 195 studies, only 84 reported the software program that was used to run their analysis. Such limitations indicate that the workflows are not reproducible. While an inability to reproduce results does not necessarily imply that the results are misleading or incorrectly calculated, it may undermine public trust in the findings. This is especially true in the context of the energy transition, where the high investment costs of ambitious policies are in the public’s interest and require a solid foundation of verifiable evidence. Furthermore, a lack of reproducibility impedes research progress by making it difficult for researchers to build on existing work.

The idea of using computational workflows to improve reproducibility in research is not new, with major contributions generally found in the life sciences, and bioinformatics in particular.^14^^,^^15^ Well-known examples include Snakemake,^16^ deepTools,^17^ and UniPro’s UGENE.^18^ Ongoing work is focused on the improvement of reproducibility and transparency in such workflows, where recent research introduces the “five pillars of reproducible computational research,”^19^ and, more recently, the argument that FAIR data principles should also apply to computational workflows.^20^

Developments include the increased use of cloud computing platforms and cluster computing, allowing computational workflows to be easily scaled and to effectively manage large datasets, which is essential for renewable energy potential analyses. Containerization further ensures portability of workflows across computing environments, for example, using Docker^21^ or Singularity.^22^ Modern workflow management tools such as Snakemake^16^ and Luigi^23^ provide graphical user interfaces for managing task dependencies and provide real-time monitoring and easy workflow adjustments.

Well-known computational workflow tools in the field of renewable energy systems include the Technical University of Berlin’s PyPSA^24^ and renewables.ninja^25^ from the Imperial College London and ETH Zürich, which has been used for estimating wind^26^ and solar PV potentials^27^ over Europe until 2030. Similarly, an open-source workflow was developed for rooftop PV assessments from satellite images in Pueblas et al.^28^ In addition, Barber et al.^29^ have developed a framework for selecting the best wind resource assessment workflow for complex terrain, which could potentially be used alongside a dedicated workflow manager tool such as the one presented in this paper. Nevertheless, an explicit workflow manager that handles all workflow steps is, to the knowledge of the authors, currently unavailable in the field of renewable energy potential analyses. A workflow manager that allows researchers to use their own input data and own models or software to conduct the analyses would thus be beneficial for improving reproducibility in the field. To address the need for improved transparency and reproducibility in renewable energy potential assessments,^4^ the authors have developed the Renewable Energy Potentials WorkFLOW tool (REFLOW), part of the “Energy Transformation PatHway Optimization Suite” (ETHOS). At a high level, all renewable energy potential analyses follow a similar conceptual framework.^30^ The conceptual framework consists of a series of tasks, which, once assigned to software, can be implemented as a workflow. The REFLOW tool provides a framework for conducting resource potential assessments in a fully transparent and reproducible manner, from data acquisition to eligibility assessments and simulations.

**Figure 1:**
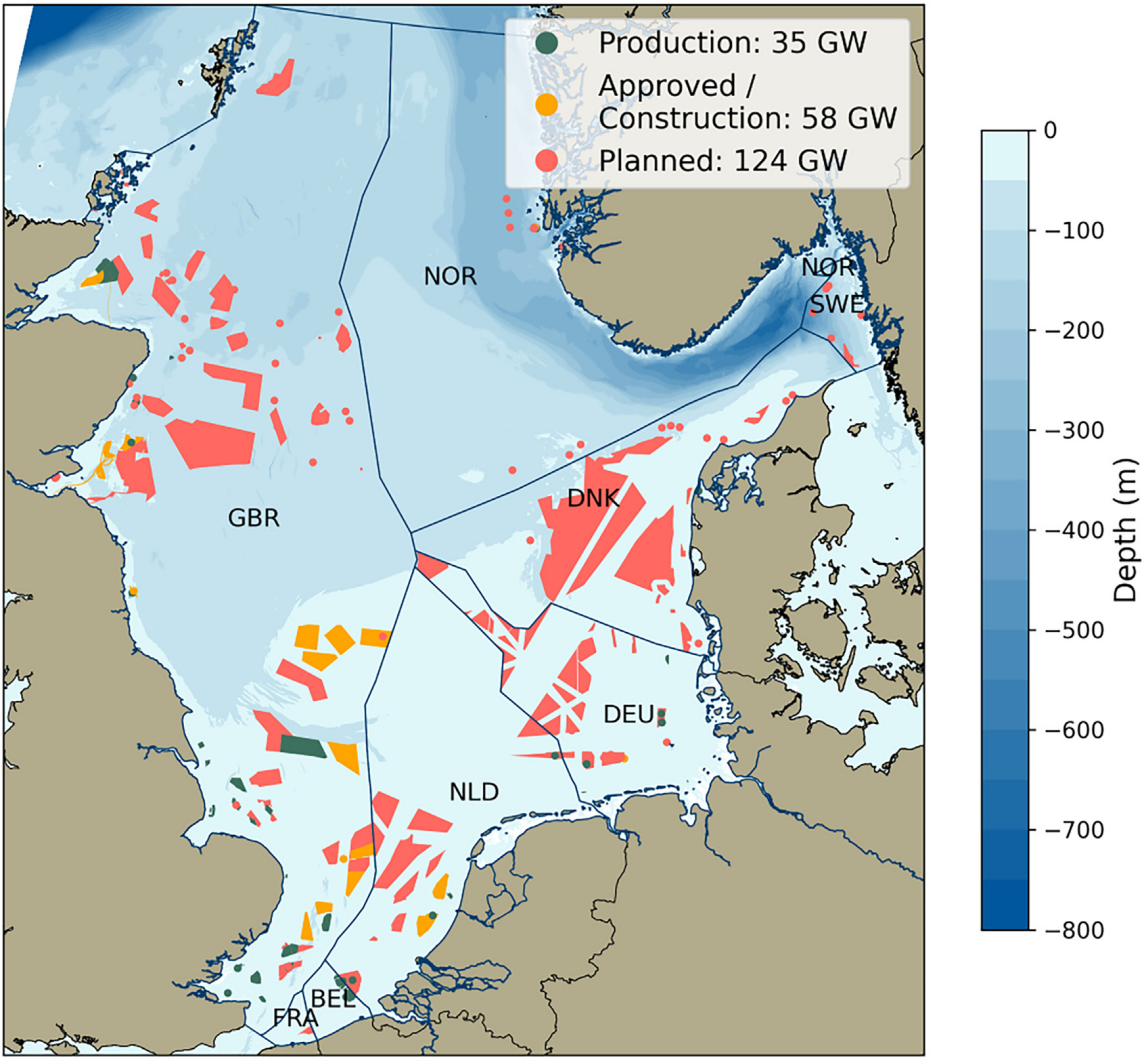
Illustration of the current wind projects in the North Sea by status, along with the bathymetry of the study region The maximum depth of <700 m occurs in the Norwegian Trench.

In this paper, we describe the development of the REFLOW workflow using a case study estimating the technical offshore wind potential of the North Sea. An additional case study for onshore wind exists on the project’s GitHub repository (see data and code availability). The North Sea is of great importance in achieving Europe’s renewable energy goals, including 300 GW offshore wind power by 2050^31^ and the REPowerEU goals^32^ (see Figure 1). Recent research on North Sea wind resources includes studies relating to future energy system modeling,^33^^,^^34^ reductions in environmental impacts through the use of larger turbine models,^35^ effects of wakes between existing wind farms,^36^ and the long-term variability of the wind resources.^37^

We identified six studies explicitly assessing the technical wind potential for the North Sea. Schillings et al.^10^ developed a tool for identifying favorable areas for wind development, and estimated a geographical potential of 31,416 km^2^ and an annual energy production potential of 259 TWh for a large area of the North Sea. Gusatu et al.^9^ estimate the region’s technical potential, with a maximum installable capacity of 1,898 GW. Sørensen and Larson^8^ perform a techno-economic analysis for offshore wind in the North Sea, estimating a maximum installable capacity of 1,200 GW. Hahmann et al.^7^ assess the technical potential of the North Sea under different wake effect scenarios for three turbine types, while Jongbloed et al.^38^ consider various spatial constraints in relation to the area available for offshore wind development. Santhakumar et al.^6^ estimate the maximum offshore development by 2030 and 2050 under various policy options. Directly comparing these studies is challenging because of the variations in their focus and reported metrics: some aim to assess the space available for development,^38^ while others estimate energy production^7^^,^^10^ or installed capacity.^8^^,^^9^ While the studies use open data, none share the input data itself, nor code for preprocessing the data or running the analysis. Thus, a reproducible workflow for the North Sea’s technical offshore wind potential is of significant interest to the renewable energy sector.

## Results

### Description of REFLOW

In renewable energy analyses, there is an important distinction between “resource assessments” and “resource potential studies.” Resource assessments typically focus on project-specific evaluations, providing highly localized and granular data on wind speed, capacity factors and site-specific considerations. Conversely, resource potential studies estimate theoretical, geographical, technical, or techno-economic potentials over larger regions, providing insights for policymaking and energy system planning. Although REFLOW is primarily designed for the latter, its modular framework lends itself well to future adaptations for project-specific resource assessments. In the sections below, we refer to “renewable potential analyses” primarily regarding larger-scale resource potentials as outlined. However, the workflow’s structure would be highly conserved for project-specific resource assessments, where it can be used to manage processes for providing individual site evaluations.

**Figure 2:**
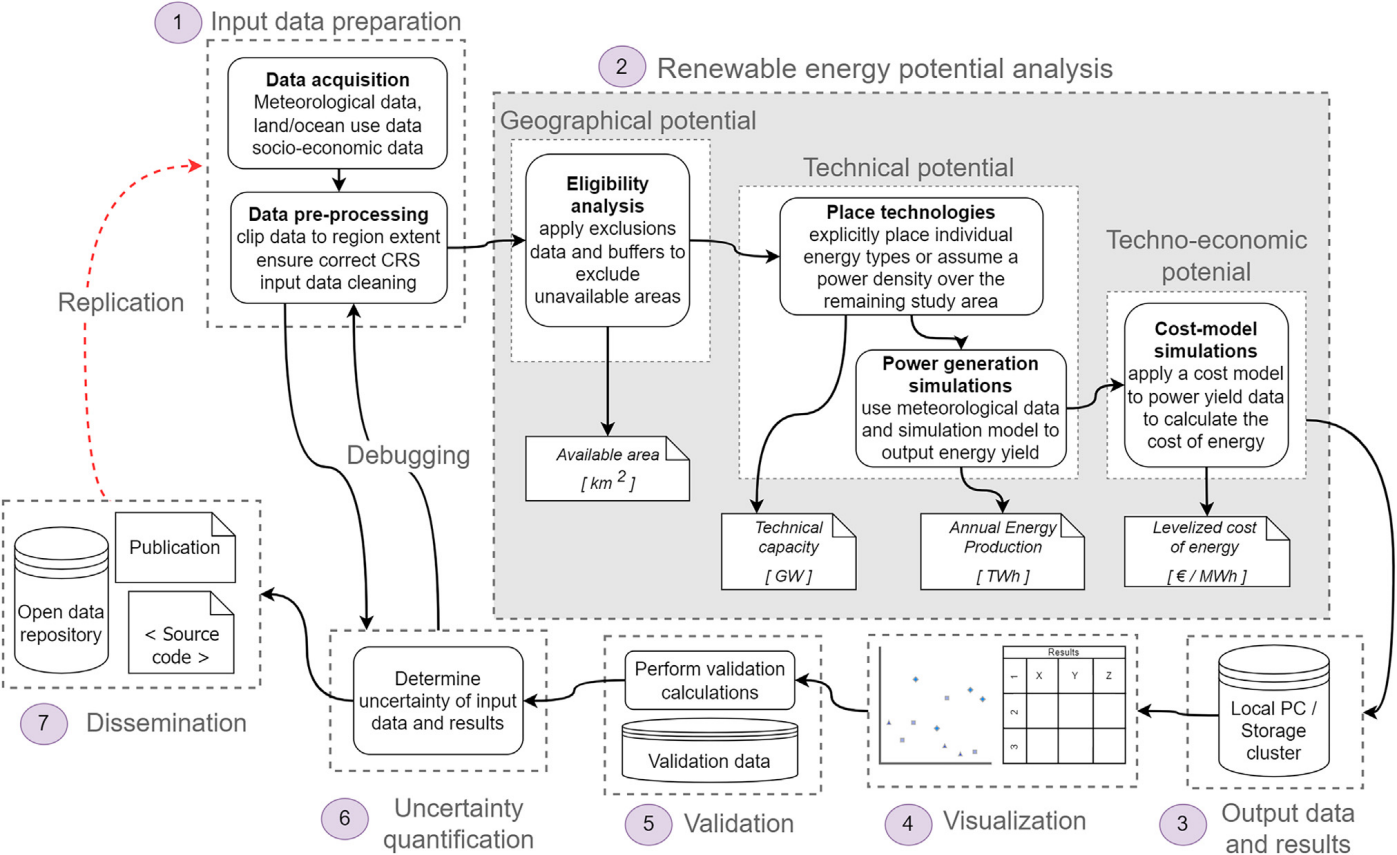
Overview of an idealized workflow for renewable energy potential analyses While many studies focus solely on geographical or technical potential^4^ (as is the case in this paper’s case study), the full workflow is illustrated here for comprehensive understanding. The flowchart includes the concept of debugging, which is necessary in computational research projects for correcting errors by iterating previous steps. The red dotted line represents the process of replication, allowing other researchers or energy system modelers to repeat the project using open data and source code. This step is often not possible due to the lack of data, code, or adequate project documentation.^4^ Figure adapted from Guo.^39^

The workflow for renewable potential analyses can be divided into seven main tasks, each encompassing multiple processes^30^ (see Figure 2). These tasks are: (1) input data preparation, (2) the renewable energy potential analysis itself, (3) output of data and results, (4) visualization, (5) validation, (6) uncertainty quantification, and (7) dissemination. These tasks are discussed in more detail in the sections below. In Figure 2, task 6 links back to the input data preparation task via the “debugging” process, an integral part of the workflow, although in an actual workflow the debugging process can link any of the tasks together. This process involves repeating various steps to resolve errors, a common aspect of all data-intensive research. The dissemination stage is linked back to the data acquisition process through the “replication” process. This step, often missing in renewable potential analyses, facilitates the replication of the study by providing access to the source code and input data.

REFLOW^40^ is a Python-based framework for managing renewable energy potential workflows, developed with the aim of automating the above workflow except for dissemination. It is built on the open-source workflow manager package Luigi^23^ and uses a dependency graph architecture for workflow orchestration and task dependency management. This allows for user-friendly management of interdependent tasks and full reproducibility of renewable energy potential assessments, from data acquisition through eligibility analyses, simulations, visualizations, and reporting.

The concept of a “task” is central to REFLOW’s structure (i.e., the tasks described in Figure 2). Tasks are defined as individual processes that can be executed by one or more software applications. Initially, each task exists as a theoretical entity that must be assigned to a specific software program for execution. REFLOW transforms these theoretical tasks into operational sequences by assigning the appropriate software and managing input and output data, enabling the practical execution of a renewable energy potential workflow. REFLOW provides a structured, reproducible framework for renewable energy potential analyses. The modular design simplifies complex workflows and supports the capacity for energy system modelers to build on previous studies. The sections below introduce the workflow components of REFLOW.

### Pre-analysis tasks

#### Software installation

A REFLOW instance initiates with an “*Environment setup*” script, which manages Python package dependencies based on specific software requirements of the project. This setup ensures smooth workflow execution. REFLOW’s design requires that all third-party software integrated into the workflow must be operatable through a Python interface. However, this requirement does not significantly limit the tool’s capabilities, as several software programs with sufficient Python-interfacing capabilities are available. A selection of these programs, which could be integrated into a REFLOW workflow, are described in Table 1.

**Table 1:** Common software used in renewable energy potential studies, which are either built in Python or have a Python interface

Name	Developer	Use	Python interface
ArcGIS^41^	Esri	geospatial analysis and mapping	ArcPY^42^
EnergyPlus^43^	U.S. Department of Energy	building energy simulations	Eppy^44^ and Geomeppy^45^ packages
Geospatial Land Availability for Energy Systems (GLAES)^46^	Forschungszentrum Jülich, ICE-2	geospatial analysis for eligibility studies	direct – built in Python
Python for Power System Analysis (PyPSA)^24^	TU Berlin, Department of Digital Transformation in Energy Systems	renewable energy system optimization and simulation	direct – built in Python
PyWake^47^	DTU Wind Energy	wake and flow field simulations	direct – built in Python
Renewable Energy Simulation toolkit (RESKit)^48^	Forschungszentrum Jülich, ICE-2	renewable energy simulations	direct – built in Python
System Advisor Model (SAM)^49^	National Renewable Energy Lab (NREL)	renewable energy simulations	pysam^50^
QGIS^51^	QGIS Development Team	geospatial analysis and mapping	PyQGIS module (part of QGIS)
WAsP^52^	DTU Wind Energy	wind resource assessments and siting	pyWASP module (part of WAsP)
Weather Research and Forecasting Model^53^	National Center for Atmospheric Research (NCAR)	high-resolution atmospheric simulations and wake modeling	wrf-python^54^

The case study presented in this paper makes use of the software Geospatial Land Availability for Energy Systems (GLAES)^46^ and the Renewable Energy Simulation toolkit (RESKit),^48^ along with the python packages built into REFLOW itself.

Following the environment creation, a series of tasks are performed in a particular order as a workflow, an example of which is illustrated in Figure 7. REFLOW is built with reproducibility in mind and, thus, to reduce the need for users to dig into the code as much as possible, all necessary variables to run a workflow are defined in a set of settings files. Running a variety of scenarios can be achieved through updating variables in one or more of the settings files. These include.

(1) Project_settings: specifies the list of countries or regions, or the bounding box extent of the study region, as well as the zoom level (national, subnational, or municipal), the project’s spatial reference system, and the start and end year for the project.
(2) Exclusions_settings: contains a dictionary of the constraints as well as the associated SQL queries and buffer values.
(3) Technologies: defines the configuration settings for the technologies being utilized, such as wind turbine specifications (hub height, capacity, rotor diameter, turbine spacing) or solar PV parameters (capacity, tilt, panel type). Can be a single technology type or multiple.

#### Data acquisition

The data acquisition task addresses an ongoing issue in renewable potential studies: making input data readily available. Even for modelers concerned with making their primary data available to ensure reproducibility, this task has challenges. Primarily, the sheer volume of geospatial and meteorological data (often several hundreds of gigabytes or more) makes dissemination through open repositories impractical. Furthermore, data rights clauses often restrict the redistribution of openly available datasets. For example, licenses such as the Creative Commons Attribution-NonCommercial^55^ and the Open Data Commons Attribution License^56^ permit the use of data for research purposes but do not allow for their redistribution.

Given these constraints, a simple approach involves providing users with scripts to automatically download the input data upon initiating the project—thereby eliminating the need to redistribute the data and still allowing for reproducibility. The obvious drawback is that the data must be openly available and accessible from a public repository or via an API request.

#### Data preprocessing

Data preprocessing, an essential step in preparing datasets for analysis, involves detecting and correcting or removing corrupt, inaccurate, incomplete, or irrelevant data. It ensures dataset quality and readiness for subsequent tasks.^57^ This includes handling missing data, cleaning, and transforming datasets. Specifically, in renewable energy workflows, data preprocessing often involves managing geospatial data (e.g., vector and raster transformations), processing time series data, calculating elevated wind speeds, interpolating meteorological data to higher resolutions, and file-type transformations. Furthermore, the IEC 61400 standards specify important steps in data collection and preprocessing for site-specific wind potential assessments, for example, on how to apply air density adjustments to wind speed values extrapolated to hub height.^58^ Despite its importance, the full workflow of data preprocessing reporting is rarely reported in publications, leading to non-reproducible analyses.^4^ To address this, REFLOW integrates data preprocessing into its workflow, providing multiple utility functions for common preprocessing steps and ensuring full reproducibility.

### Renewable energy potential analysis

Renewable energy potential studies typically perform an eligibility analysis of the study area to ensure that non-available areas are not assumed to be available for development.^4^^,^^5^ There are two approaches to this task, the most common being a binary approach where pixels are considered either available or unavailable for development.^4^^,^^5^^,^^59^ This is typically done through a “greenfield” approach, where undeveloped land (or ocean area) is prioritized.^59^ In other studies, a “brownfield” approach is adopted, where existing sites can be upgraded—for example, in the case of rooftop solar PV.^60^ The second possibility is to use a multi-criterion decision analysis (MCDA) tool to weight various criteria and produce areas with varying degrees of suitability, based on an optimization metric.^61^^,^^62^ This type of analysis allows for the incorporation of viewpoints of various stakeholders, including social barriers and uncertainties, and is sometimes referred to as the “feasible potential” of a region. While the case study below presents a technical potential analysis, an MCDA-based approach could also be implemented using REFLOW as a workflow manager.

Next, the available area is populated with the given technologies used in the study: for example, specific types of onshore wind turbines or solar PV devices. In many analyses, this is done by assuming a power density over the available area, for example in MW/km^2.^^30^ In others,^48^^,^^63^ technologies are explicitly placed over the study area at designated coordinates. In this analysis, we employ both approaches over the North Sea.

### Post-analysis tasks

In renewable energy potential assessments, validation typically involves comparing model outputs with real-world data, such as operational data from wind farms or solar PV sites, to quantify model output errors. However, this process is often hindered by limited access to high-quality operational data that are generally proprietary. In such cases, validation can only be performed with respect to the results of existing literature. Furthermore, project uncertainty can stem from various sources, including input data quality, model limitations, spatial and temporal resolution, and assumptions from the research team conducting the analysis.^26^^,^^64^^,^^65^^,^^66^ Quantifying this uncertainty is particularly important in renewable energy assessments, given the large capital expenditures and the complexities of integrating variable renewable energy into existing energy systems. To address these challenges, REFLOW allows users to incorporate additional scripts, such as Monte Carlo simulations or sensitivity analyses, to assess the impacts of input variability on results. REFLOW also enables the integration of multiple methodologies within a single workflow, allowing users to explore how different approaches influence outcomes, thereby enhancing the robustness and transparency of renewable energy potential assessments.

A key strength of the tool is its capacity to streamline workflow management. Where open data are used, renewable energy potential analyses often inadequately describe the preprocessing steps or do not share the underlying code base, making replication nearly impossible.^4^ REFLOW overcomes this by managing the execution of the entire workflow through a single script, supported by comprehensive debugging logs for each task. By integrating container software such as Docker,^21^ REFLOW enables an entire study to be reproduced from start to finish with a single line of code. Built on Luigi,^23^ it also supports parallel task execution using clusters or multiple CPUs. In addition, REFLOW enhances transparency by generating detailed output logs and machine-readable JSON reports, which can serve as inputs to energy system optimization models, such as in Groß et al.^67^ Together, these features make REFLOW a useful tool for improving transparency, data management, and reproducibility in renewable energy potential analyses.

## Case study results

Below we present the results of the North Sea case study. Note that these scenarios are hypothetical and represent the maximum technically feasible wind power deployment and not actual national plans for development.

### Geographical potential

The initial available area for wind park development in the North Sea is 559,452 km^2^. Excluding boundaries of 12 nautical miles from coastlines reduces the available area by 20%. For the 50 m depth scenario, the ocean depth is the greatest impact on the available area—accounting for just under 60% of the total exclusions. The remaining exclusions fall under the category of “human activities” and are outlined in Table 2. Of these, active oil and gas exploration licenses have the greatest impact, excluding 21.5% of the total area, followed by protected areas at 13.8%.

**Table 2:** Exclusions and buffers employed in this study

Exclusion	Distance (m)	Data provided by
**Physical**		

Minimum distance from coast	22,224 (12 nautical miles)	Maritime Boundaries Geodatabase^85^
Maximum ocean depth	50 (shallow scenario) 1,000 (deep scenario)	GEBCO^71^

**Human activities**		

Cultural heritage	100	EMODnet Human Activities^72^
Desalination plants	200	
Dumped munitions	300	
Energy projects	500	
Marine protected areas	1,000	
Military areas	400	
Oil and gas licenses	0	
Power cables	500	
Shipping lanes	500	
Telecom cables	500	
Wind projects: approved/in service	1,500	

**Figure 3:**
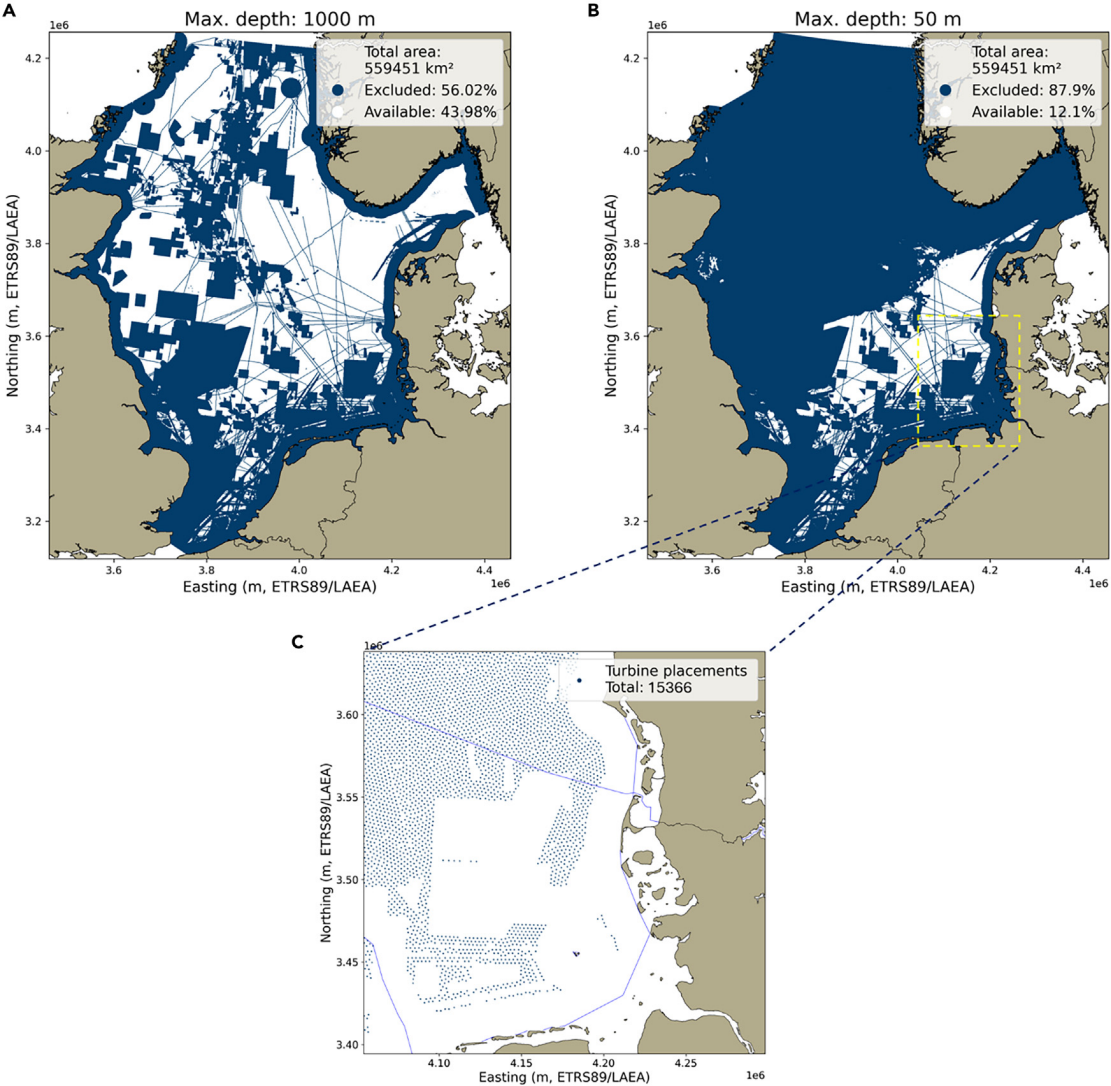
Remaining area for development after excluding non-available areas due to human activities in the (A) 1,000 m depth scenario and (B) 50 m depth scenario; and (C) a zoom area off the German and Danish coasts, illustrating the turbine placement algorithm

When accounting for all exclusions, the remaining area in the 50 m depth scenario is reduced by 88% (491,765 km^2^), as illustrated in Figure 3. For the 1,000 m depth scenario, 56% (313,397 km^2^) of the area is excluded. Thus, the *geographical potential* for the North Sea is 67,686 km^2^ for fixed-foundation turbines, and 236,054 km^2^ for fixed-foundation and floating-turbine technology.

### Technical potential

Assuming a turbine matching the characteristics as the largest currently available offshore turbine in Europe, the Vestas V236-15MW, and a spacing of 2,360 m between each turbine (10× rotor diameter of 236 m), a total of 15,366 turbines can be sited in the 50 m scenario, and 51,212 turbines in the 1,000 m depth scenario. With a nameplate capacity of 15 MW, the total installable capacity for the explicit placement method is 230 GW (50 m depth) or 768 GW (1,000 m depth). In the uniform power density method, we multiply the available area by the power density in MW/km^2^, resulting in a slightly higher installable capacity of 238 and 861 GW for the fixed-foundation and mixed-technology scenarios, respectively. National-level geographical potentials and installable capacities can be found in the supplemental information.

**Figure 4:**
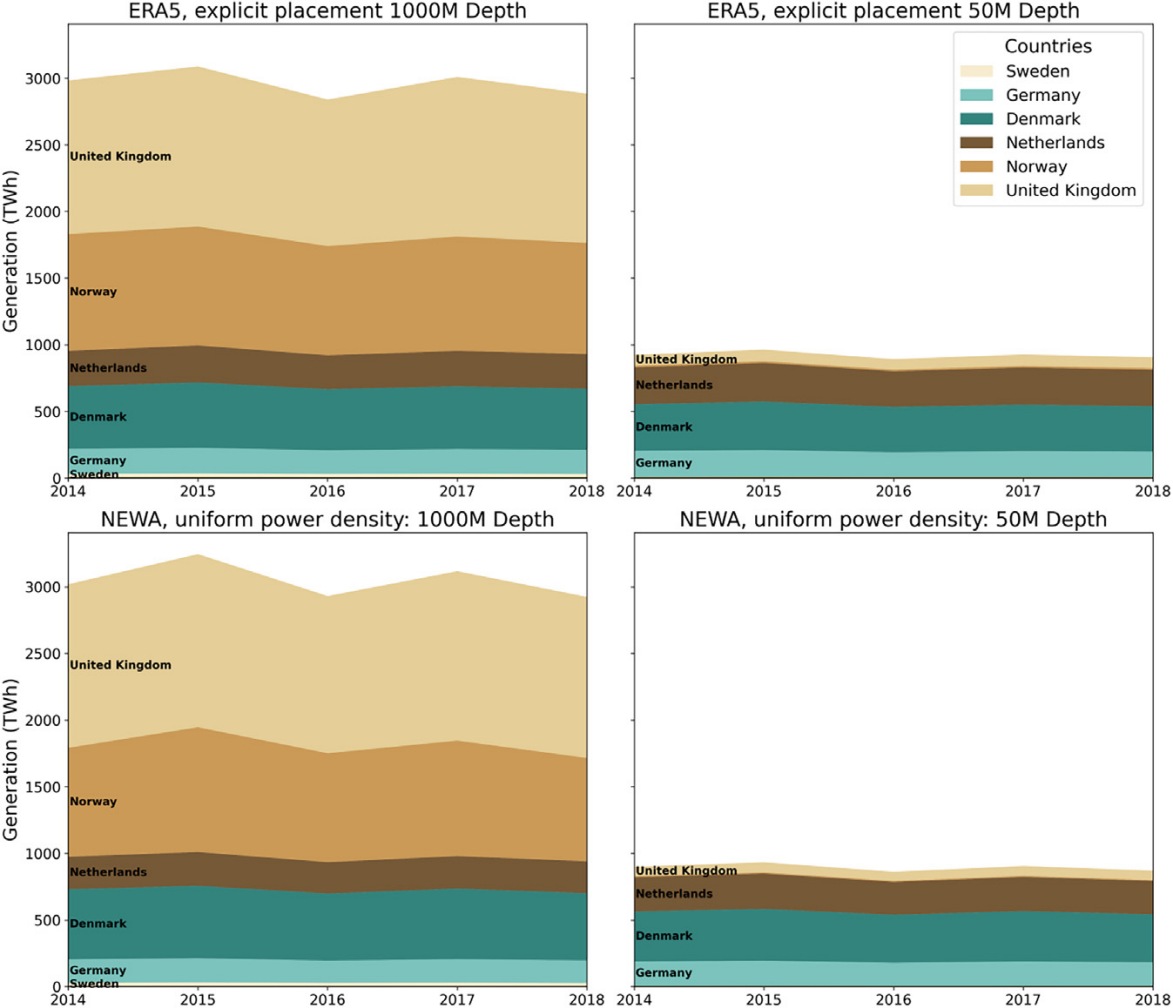
Annual generation (TWh) for each country over 2013–2023 Note that Belgium and France are not included in the plot legend as their representative area is too small to be seen. The interannual variability can be seen, especially for 2015 and 2017, where annual energy yield is particularly high for the mixed-technology scenario across both methodologies.

For the explicit placement method, simulated with ERA5 reanalysis data, the mean energy yield for the total North Sea area is 923 and 2,961 TWh for the fixed-foundation and mixed-technology scenarios, respectively. For the uniform power density method, incorporating NEWA time series data, the generation is slightly higher at 894 and 3,047 TWh for the two technology scenarios. The total annual generation over the study period is illustrated at a national level in Figure 4. The greatest national contributors to the overall generation capacity are the UK, Norway, and Denmark, for the mixed technology scenario, and the Netherlands, Denmark, and Germany for the fixed-foundation scenario.

**Figure 5:**
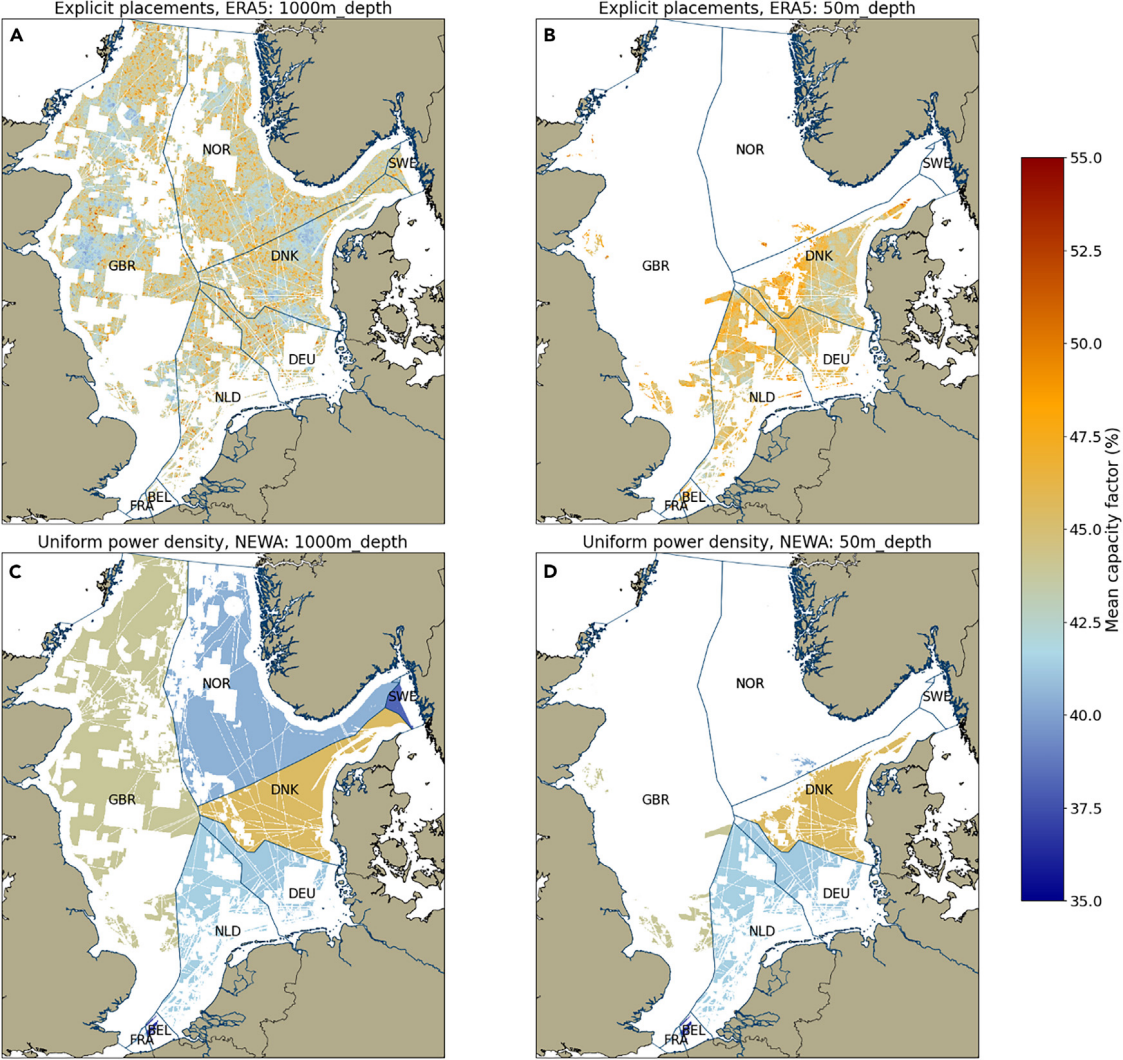
Spatial variability of capacity factors over the North Sea Spatial distribution of capacity factors (A and B) and standard deviation, representing variability (C and D) over the study period 2013–2023 for each turbine placed. Note that, due to the high resolution of this panel, it may not display properly when viewing in print. When viewing online, click the image to view in high resolution.

For the ERA5 explicit placements method, the overall mean capacity factor across the 2014–2018 period is slightly higher for the fixed-foundation scenario than the mixed-technology scenario, at 46% and 44%, respectively. This is likely due to the far greater area available in the mixed-technology scenario, where areas with both better and worse conditions will be available for development. There does not appear to be a significant trend for the spatial distribution of capacity factors, with high-performing areas clustered around the entire region, particularly toward the north and central regions. For the NEWA uniform power density method, we use a mean wind speed over the entire area for each country at each time step to determine the capacity factor. Thus, the mean capacity is 41% for both technology scenarios, as illustrated in Figure 5.

**Figure 6:**
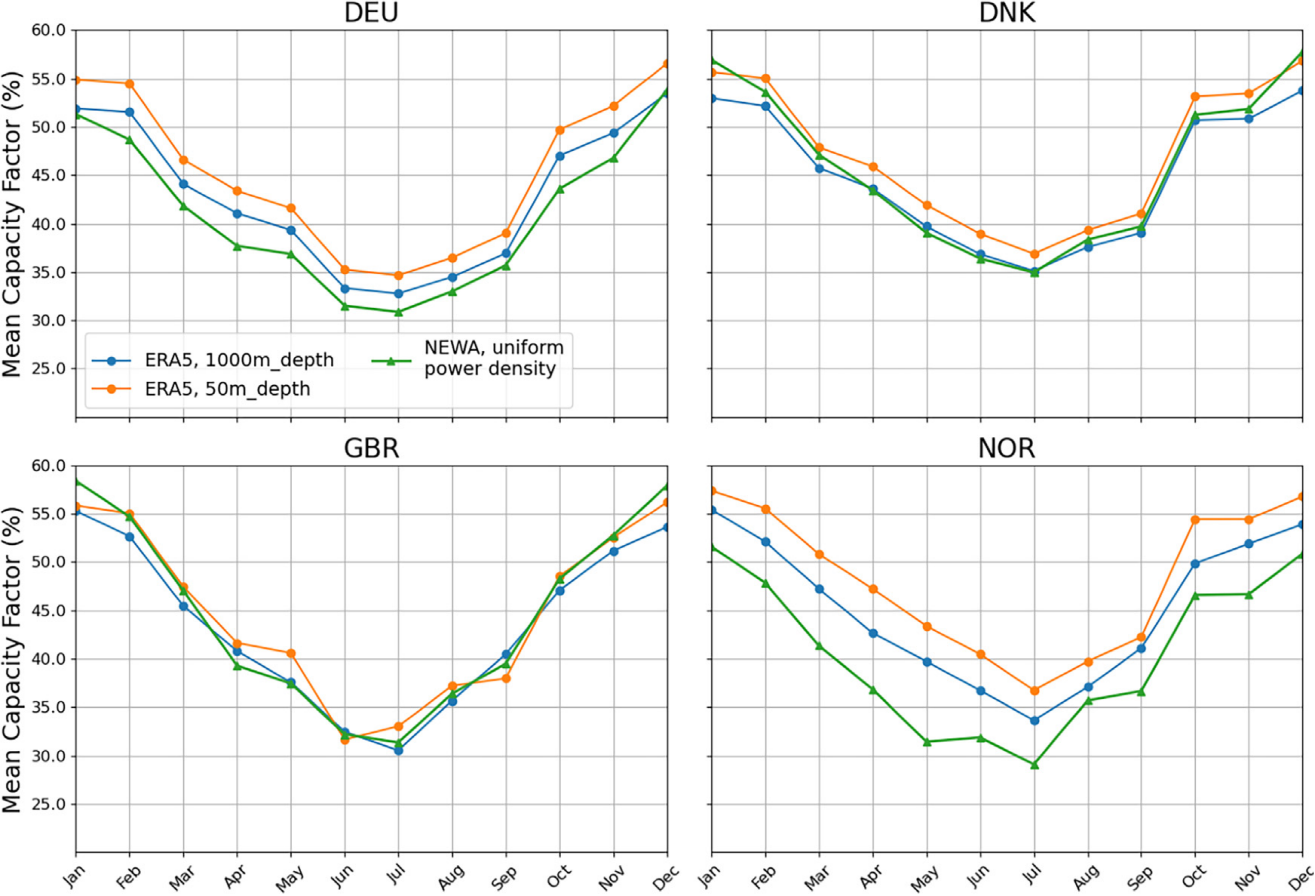
Variability of monthly capacity factors across the North Sea for the period 2014–2018 for Germany (DEU), Denmark (DNK), Great Britain (GBR), and Norway (NOR), for both study methodologies Note that the mean capacity factor is taken over the entire national EEZ for the uniform power density method, and thus is the same for both the fixed-foundation and mixed-technology scenarios.

In addition, there is a clear seasonal variability in capacity factors (see Figure 6), which is already well described in the literature.^68^^,^^69^^,^^70^ The winter months (December, January, and February) have capacity factors of around 50%–60%, while the summer months average 30%–35%. In years with reduced wind resources, such as 2014 and 2018, the summer month mean capacity factors can drop well below 30%. Thus, the wind resource over the North Sea is subject to strong inter- and intra-annual variability, which is essential to account for in energy system modeling and energy planning.

## Discussion

### Summary of key findings

In this study, we describe REFLOW and apply it in a case study to estimate the technical offshore wind potential for the North Sea under two scenarios: one using fixed-foundation wind turbines limited to 50 m depth and another incorporating fixed-bottom and floating turbine technologies. We apply two distinct methodologies to demonstrate how the tool can be used to compare study setups. A key result of this study is the demonstration of the REFLOW tool as a Python-based workflow manager for reproducible renewable energy potential analyses. REFLOW automates the workflow for large-scale assessments, from data acquisition and pre-processing through to visualizations and reporting.

The initial area available for wind park development in the North Sea (559,452 km^2^) is significantly reduced in both technology scenarios. In the fixed-foundation scenario, the geographical potential decreases by 88%, leaving 67,661 km^2^ available. Ocean depth constraints are the primary exclusion factor, accounting for nearly 60% of the reductions. In the mixed-technology scenario, the remaining area expands to 235,850 km^2^, with human activities—particularly oil and gas activities and protected areas—as the most significant exclusions.

Two methodologies were compared for evaluating the technical potential of the North Sea: explicit turbine placement and uniform power density. In the explicit placement method, turbines were modeled on the Vestas V236-15MW, with a rotor diameter of 236 m and a 10× rotor diameter spacing, with wind speed time series data from ERA5. Under the fixed-foundation scenario, an installable capacity of 230 GW and a mean annual yield of 923 TWh. In the mixed-technology scenario, the number of turbine installations increases significantly, yielding an installable capacity of 768 GW and a mean annual yield of 2,961 TWh. In the uniform power density method, a density of 3.5 MW/km^2^ is assumed and the installable capacities are slightly lower, at 238 and 861 GW for the fixed-foundation and mixed-technology scenarios, respectively. Simulating power generation based on a mean capacity factor for each country with wind speed data from NEWA results in 894 and 3,047 TWh, for the two technology scenarios.

The case study highlights a key benefit of REFLOW in terms of enabling comparisons between study setups and workflows. By automating processes such as data acquisition, pre-processing, turbine siting, and reporting, REFLOW provides a reproducible framework for transparent, reproducible renewable energy potential assessments.

### Limitations

#### REFLOW tool

The REFLOW tool simplifies the process of reproducing renewable energy resource assessments by explicitly defining a computational framework to run each task in order, in such a way that analyses can be easily rerun or built upon by editing settings rather than having to implement significant changes in the underlying code. Nevertheless, there are several limitations that should be mentioned.

REFLOW relies heavily on external applications for executing tasks. While several built-in utility functions, particularly for data pre-processing and reporting, are available to the user, key elements of the potential assessment must be run by interfacing with external software. This has the potential to cause problems with reproducibility if there are dependencies with specific software versions, or if software compatibility issues arise, which could lead to runtime errors or require extensive debugging. However, this limitation can be avoided by containerizing each workflow such that the exact computing environment is replicated during initialization.

The computational requirements of workflows may vary significantly depending on the size of the study region, the length of the simulation, the data preprocessing steps, and the simulation methodology. While REFLOW supports parallel processing, this may not be feasible with all software integrations, and performance is highly dependent on the available computational resources. Users without access to high-performance resources may experience difficulties executing complex workflows or estimating the potentials of large regions.

The reliability on Python as the programming language and Luigi as the workflow manager tool may pose challenges in terms of adaptability and an initial learning curve. While the tool is designed to make reproducible workflows as simple as possible to run, initiating a new workflow may require significant customization, especially if integrating additional software (e.g., from Table 1). Furthermore, the interdependency of tasks means that errors generated early on may be propagated through the entire workflow if not properly debugged. However, this risk is also present in renewable resources potential analyses, which do not make use of an automated workflow management tool.

As with all data-based models, the accuracy of the results of an analysis conducted with REFLOW are contingent on the input data, which may have varying degrees of accuracy and completeness. The reproducibility of a given analysis also depends on the accessibility of the input data. While many high-quality open-source datasets exist for, for example, wind speed and direction, land use categorization, and topography, renewable energy studies often depend on proprietary or restricted datasets—even more so in the case of wind or solar PV resource assessments for commercial projects. This would limit the capacity of the tool to be used for transparent, reproducible analyses.

#### North Sea case study

While the case study provides a comprehensive estimation of the technical offshore wind potential of the North Sea, several limitations should be acknowledged. First, the exclusion criteria and buffers are applied uniformly across all exclusive economic zones (EEZs) and may not reflect national or local variations in policies or siting preferences. An in-depth analysis that integrates local preferences and regulations would be valuable. The binary exclusion methodology simplifies the complexities of real-world regulations, siting, and permitting. The use of a multi-criteria decision analysis approach would be useful for including relevant stakeholders and local concerns. In addition, while we account for seasonal and interannual variability, extreme weather events and long-term climate change impacts are not explicitly considered. The study uses a single turbine configuration (Vestas V236-15MW), which may limit the range of potential results as turbine technologies develop a more nuanced approach, as in Caglayan et al.,^63^ would beneficial.

We employ a constant turbine availability factor of 92% for the fixed-foundation scenario and 87% for the mixed-technology scenario, along with an array efficiency of 88% for both scenarios to account for wake losses. However, turbine availability is influenced by complex site-specific factors including distances from shore, failure rates, repair times, ocean conditions, and maintenance strategies, while wake losses are similarly highly complex. While detailed modeling of these factors is beyond this paper’s scope, the use of constant availability and array efficiency values for all turbine locations is a limitation. Finally, this study does not comprehensively address social and economic impacts or perform a cost analysis to estimate the cost of energy, as in Santhakumar et al.,^6^ Sørensen and Larsen,^8^ and Schillings et al.,^10^ as this is outside of the current scope.

### Comparison to similar studies

A thorough validation of the results is beyond the scope of this case study. Nevertheless, a brief comparison to existing literature on the maximum wind potential of the North Sea is outlined below and in Table 3.

**Table 3:** Comparison of annual energy yield simulation methodologies used in the North Sea offshore wind potential case study

	ERA5, explicit placements method	NEWA, uniform power density method
Turbine distribution method	individual turbines explicitly distributed across remaining area	a uniform power density is applied to the remaining area
Turbine distribution software	GLAES	N/A
Power density (MW/km^2^)	∼3.3 (variable)	3.5
Wind data source	ERA5 time series^81^ interpolated with NEWA long-term mean^82^	NEWA time series^82^
Wind data height	100 m	100 m
Simulation period	2014–2018	2014–2018
Vertical extrapolation method	logarithmic law, roughness = 0.0002	logarithmic law, roughness = 0.0002
Simulation scope	capacity factor simulated for each wind turbine location	mean capacity factor simulated over entire national EEZ region

Jongbloed et al.^38^ estimate the geographical potential for approximately 75% of the North Sea, identifying 18,000 km^2^ as economically feasible for offshore wind development. However, their methodology includes additional economic constraints, making a direct comparison challenging. Sørensen and Larsen^8^ evaluate the area required to meet Europe’s electricity demand with different technology configurations, estimating that 180,000 km^2^ could fulfill a demand of 3,500 TWh with an installed capacity of 1.2 TW. Similarly, Santhakumar et al.^6^ estimate the maximum deployment of 498 GW in the North Sea by 2050, based on eight renewable energy clusters. These studies differ from ours in that they do not strictly estimate the technical potential of the North Sea and do not apply exclusions aside from the ocean depth.

Studies by Gusatu et al.^9^ and Hahmann et al.^7^ align more closely with our methodology, including scenarios for fixed-foundation and mixed-technology turbines. Schillings et al.^10^ focus exclusively on fixed-foundation turbines, limiting depth to 50 m and distance offshore to 70–150 km per country. These assumptions, combined with a low power density of 2 MW/km^2^, explain the lower estimates of geographical potential, installable capacity, and mean energy yield.

Differences in results arise primarily from the selection of exclusion criteria, buffer sizes, and power density assumptions. For example, power density varies from 2 MW/km^2^ (Schillings et al.^10^) to 6.4 MW/km^2^ (Santhakumar et al.^6^), significantly influencing the installable capacity. Our study simulates a large turbine with a high nameplate capacity of 15 MW but has a relatively low power density (∼3.3–3.5 MW/km^2^) due to turbine spacing. The hub height, wind extrapolation methods, input data, and assumptions regarding turbine availability and array efficiency also affect the results. We focus on a near-future turbine design with a 143-m hub height, using ERA5 reanalysis data and high-resolution wind speeds from NEWA. In contrast, Hahmann et al.^7^ use calibrated NEWA wind speed time series and explicitly model wake effects but not loss factors for turbine availability, which explains the higher capacity factors and mean power yield. The mean capacity factor in our study ranges from 41% to 46%, lower than in the other studies. This is likely due to the larger turbines employed in our study and the conservative loss factors applied for turbine availability and array efficiency.

### Conclusions on reproducibility and transparency

Reproducibility and transparency are fundamental to the scientific method. Since most renewable energy potential studies are performed by researchers in a scientific setting, these facets should be applicable to the analyses. By automating the entire workflow, from data acquisition and preprocessing to simulation and reporting, REFLOW helps ensure that renewable energy potential studies become transparent and accessible. Each task in the workflow can be altered with the use of settings files, which means that sensitivity analyses or variations on scenarios can easily be performed.

REFLOW enables academic researchers, energy system modelers, and policymakers to build on existing studies using accessible code and comprehensive documentation, for example, in the output logs. Thus, researchers using REFLOW do not need to “reinvent the wheel” each time a potentials analysis is performed. As an example, updated data sources can easily be integrated into existing workflows, ensuring that the assessment remains up-to-date and relevant. The REFLOW tool provides a framework for overcoming the challenges relating to reproducibility, and thereby helps strengthen public trust in findings, and helps further develop the robustness of energy system models, which are essential for achieving secure future energy systems.

## Methods

**Figure 7:**
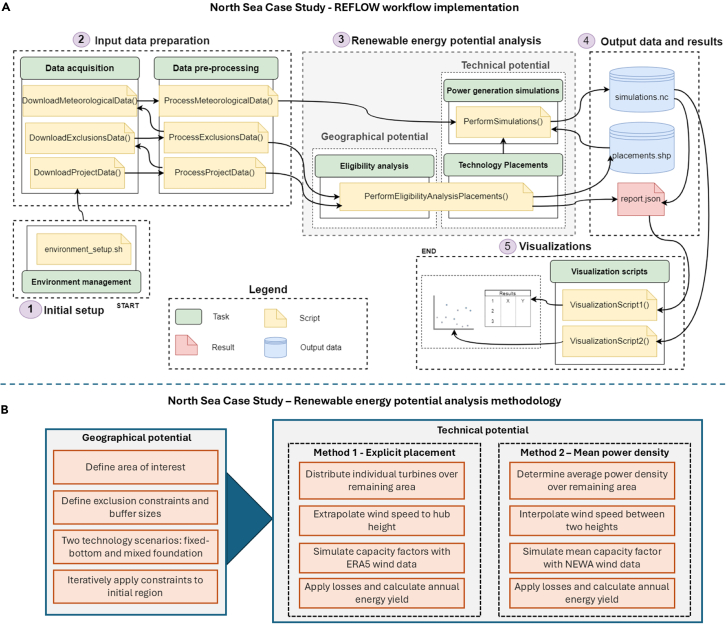
Viusalization of the study workflow (A) Illustration of the REFLOW workflow dependency graph and ordering for a simple workflow estimating the technical potential of the North Sea. Luigi^23^ acts as the primary workflow manager. Tasks are represented as rectangles, with the primary task name on top and the Luigi task name(s) printed beneath. Settings files handle all important information for each task and allow the user to easily update changes without altering the main code base. The primary outputs of the workflow are the report.json files; (B) flowchart representation of the renewable energy potential part of the case study.

To demonstrate REFLOW in detail, a case study estimating the technical offshore wind potential of the North Sea is presented in this article. Although specific datasets and methodologies (e.g., exclusion criteria, buffer sizes, wind speed extrapolation methods, or geospatial data interpolation) may vary between projects, the fundamental workflow structure is conserved. In this case study, we compare two methodologies for estimating energy yield across two scenarios: a fixed-foundation scenario (maximum depth 50 m) and a combined bottom-fixed and floating technology scenario (maximum depth 1,000 m). The latter is set to the maximum depth for floating turbine anchors, while the actual maximum depth of the North Sea does not exceed 750 m.^71^ The two methodologies employ different wind speed datasets and distinct approaches to turbine siting and energy yield simulations, allowing for a comparison of their impacts on the technical potential. The methods and scenarios are described in detail in the sections below, and an overview of the workflow is presented in Figure 7.

### Available area and installable capacity

A set of high-quality datasets are employed to facilitate the exclusion of areas not available for wind development and for simulating wind power generation (see Table 4). Exclusion-relevant data include national and maritime boundaries, such as EEZ delineations, bathymetric data, and several datasets on various human activities in the North Sea, provided by the European Marine Observation and Data Network (EMODnet).^72^ These data are open to the public and have a high degree of coverage and accuracy.

**Table 4:** Sensitivity analysis for exclusion types on the remaining area available for wind park development (geographical potential)

Exclusion	Area (km^2^)	% excluded
Initial area of North Sea polygon	559,452	N/A

**Physical**		

12 nautical miles from coastlines	111,540	19.9
Maximum ocean depth		
1,000 m	0.0	0.0
50 m	327,078	58.5

**Human activities**		

Oil and gas activities	120,095	21.5
Protected areas	77,016	13.8
Shipping lanes	47,886	8.6
Pipelines	27,931	5.0
Telecommunication cables	21,173	3.8
Military areas	16,986	3.0
Existing wind farms	12,530	2.2
Power cables	5,715	1.0
Dumped munitions	191	0.0
Ocean energy projects	108	0.0
Shipwrecks	17	0.0
Desalination plants	1	0.0

We employ the open-source Python package, Geospatial Land Availability for Energy Systems (GLAES),^46^ to exclude non-available areas from the project area. We exclude a minimum distance from the coast of 12 nautical miles (22.2 km), according to international regulations surrounding free travel of ships through these areas.^7^^,^^73^ Assuming that wind projects will be developed in the near future, we apply a maximum ocean depth of 1,000 m, suitable for floating turbine installations. We do not exclude areas with a minimum mean wind speed as the North Sea is well known for its consistently high wind speeds and is not subject to topographical interference as with onshore installations.

Human activities in the North Sea, such as active wind project sites, military zones, and marine protected areas, are mapped using the EMODnet database.^72^ The full set of exclusions and buffers is provided in Table 5. We exclude areas for existing wind project licenses that are approved, under construction, or currently in production, totaling 93 GW capacity at the time of publication (according to the EMODnet data^72^). For these projects, we apply a buffer distance of 1,500 m. Planned projects, totaling 124 GW capacity, are not excluded, as they remain open for development.

**Table 5:** Comparison of results for the maximum technical wind potential offshore North Sea against results from the literature

Reference	Max. depth (m)	Power density (MW/km^2^)	Hub height (m)	Geographic potential (km^2^)	Installable capacity (GW)	Technical potential (TWh/year)	Avg. capacity factor (%)
Schillings et al. 2012^10^	50	2	90	31,416	87	253	53
Gusatu et al. 2020^9^	55; no constraint	6.4	N/A	52,640; 296,586	336; 1,898	N/A	N/A
Hahmann et al. 2023^7^	1000	4	89–150	244,541	970	4,503	50–60
Santhakumar et al. 2024^6^^,^^a^	55; no constraint	6.4	N/A	N/A	222; 498	N/A	55–60
This study, 2024	50; 1000	3.5	143	67,686; 246,054	238; 861	894; 3,047	41–47

Where multiple scenarios are used, these are separated by commas. In this table, we compare the maximum potentials from each study. For Hahmann et al., we compare the scenario with a 4 MW/km^2^ deployment and meso-wake modeling.

a Does not include the full North Sea and rather estimates offshore wind deployment alongside additional renewable energy deployments for eight deployment areas up to 2050.

The case study applies uniform exclusion criteria and buffer distances across the North Sea’s EEZs, aligning with similar methodologies in previous studies (e.g., Hahmann et al.,^7^ Schillings et al.,^10^ Doljak et al.,^61^ Caglayan et al.,^63^ Flanders Marine Institute,^74^ and Arent et al.^75^). While this approach simplifies the analysis, it may overlook specific national siting policies and local socio-economic preferences. A more detailed, region-specific analysis is beyond the scope of this study, as our primary aim is to demonstrate the capabilities of the REFLOW tool.

Social concerns related to offshore wind developments often focus on visual impacts and the preservation of coastal landscapes.^76^^,^^77^^,^^78^ By implementing a 12 nautical mile exclusion zone from the coast, we significantly reduce the impact of such concerns. Moreover, potential concerns over impacts on marine wildlife,^79^^,^^80^ which may differ regionally, are mitigated by applying extensive buffers around marine protected areas in the North Sea.

### Annual energy yield simulations

In this section, we employ two methodologies for estimating the mean annual energy yield for the North Sea, both of which are run by REFLOW. In the *explicit placement method*, we explicitly place individual turbines over the remaining study area and then simulate the capacity factor for each turbine with data from ERA5.^81^ In the *uniform power density method*, we apply a uniform power density value of 3.5 MW/km^2^ over the entire remaining area and simulate the mean capacity factor for each country’s EEZ area with 30-min time series wind speed data from NEWA.^82^ The two methodologies are compared in Table 6 and described in more detail below. We employ a synthetic power curve based on a Vestas V236-15.0 turbine^83^ with a rotor diameter of 236 m and a nameplate capacity of 15 MW, for both methodologies.

**Table 6:** Datasets employed in the North Sea case study

Provider	Dataset name	Version	Use	File type
**Datasets used for excluding non-available areas**				

GADM^84^	none (national borders*)*	v4.1	identification of national borders	ESRI Shapefile
Flanders Marine Institute^85^	Maritime Boundaries and Exclusive Economic Zones (200 NM)	2023	identification of national EEZ boundaries	ESRI Shapefile
Flanders Marine Institute^85^	North Sea IHO Region & Skagerrak Channel	2023	define the working region for the project	ESRI Shapefile
General Bathymetric Chart of the Oceans (GEBCO)^71^	Gridded Bathymetry Data	GEBCO_2023	exclusion of areas exceeding maximum ocean depth	GeoTiff
European Marine Observation and Data Network (EMODnet)^72^	EMODnet Human Activities	2024	exclusion of sea area categories not available for wind project development	Geodatabase

**Datasets used for wind power generation simulations**				

Corpernicus Climate Data Store^81^	ERA5 Single Levels Hourly Reanalysis	2024	hourly timeseries wind data	Netcdf4
New European Wind Atlas^82^	New European Wind Atlas (NEWA)	2024	high-resolution long-run average wind speed; 30 min time series wind data	Geotiff; Netcdf4

#### Explicit placement method

Once the non-available areas have been excluded, REFLOW then distributes turbines over the remaining area using GLAES.^46^ We apply a turbine spacing of 10 times the rotor diameter, as in described in the literature.^10^^,^^86^^,^^87^^,^^88^^,^^89^ Within the North Sea, there are multiple wind patterns and thus the application of a more nuanced approach to turbine spacing, where the axial wind direction is considered, is only possible at a smaller spatial scale: i.e., the wind farm scale, which is outside the scope of this assessment.

The Renewable Energy Simulation ToolKit (RESKit),^48^ an open-source Python package, then simulates wind power generation for each turbine over an 5-year period from January 2014 to December 2018 by fitting the turbine’s power curve to the wind speed data using reanalysis data from ERA5^81^ bilinearly interpolated to a higher spatial resolution using climate mean data from the New European Wind Atlas (NEWA).^82^ ERA5 is often used in wind potential assessments^4^^,^^5^ but has a relatively low resolution of ∼31 km, while NEWA is a recent dataset based on mesoscale atmospheric simulations from the Weather Research and Forecasting model over 30 years, downscaled to 3 km spatial resolution.

#### Uniform power density method

**Figure 8:**
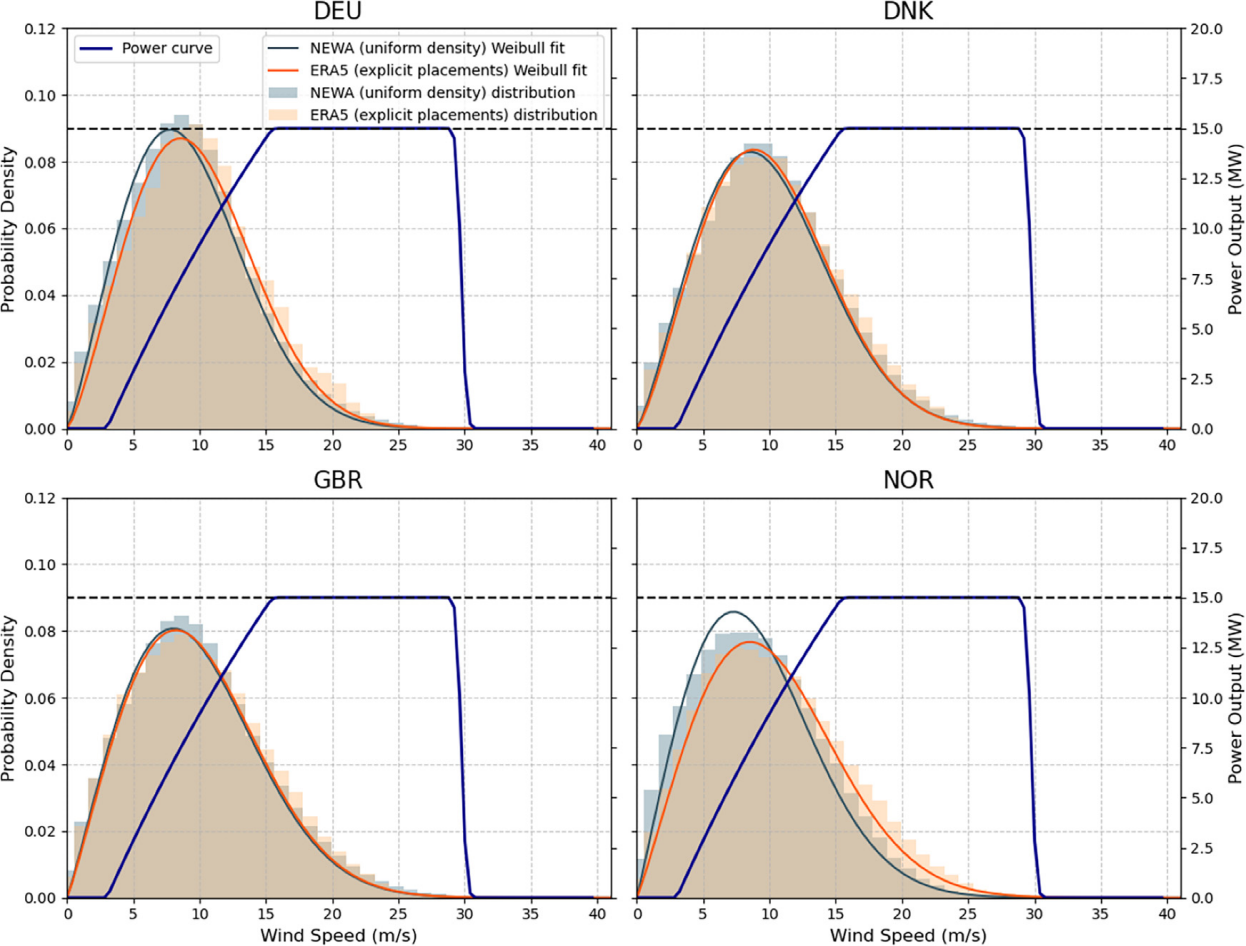
5-year wind speed distribution at 143 m hub height with fitted Weibull curve for Germany, Denmark, UK, and Norway, plotted against the synthetic turbine power curve, for both methodological approaches—explicit turbine placements with ERA5 data and a uniform power density approach with NEWA time series wind data

The installable capacity is estimated by multiplying the remaining available area by a uniform power density value of 3.5 MW/km^2^.^30^ This approach simplifies the calculation by assuming an even distribution of turbines across the entire area, and is commonly used in wind potential assessments.^4^^,^^5^ The power yield is then simulated as in the explicit placement method for each country’s EEZ, as illustrated in Figure 8.

Prior to simulating the power generation, we use the logarithmic law^90^ to extrapolate the wind speed from the reference height *r* (in this case, 100 m) to the hub height *h* of 143 m, with an assumed surface roughness factor $z_{0}=0.0002$ (Equation 1).

(Equation 1) $$V_{h}=V_{r}\left(\frac{\ln\left(\frac{h}{z_{0}}\right)}{\ln\left(\frac{r}{z_{0}}\right)}\right)$$

where $V_{h}$ is the wind speed at hub height, $V_{r}$ is the reference wind speed at height *r*, $z_{0}$ is the surface roughness length.

As per IEC 61400 guidelines,^58^ an air density correction is applied to the wind speeds based on the boundary layer height, surface pressure and surface air temperature, based on Picard et al.^91^ (Equation 2).

(Equation 2) $$\rho =\frac{pM_{a}}{ZRT}\left(1-x_{v}\left(1-\frac{M_{v}}{M_{a}}\right)\right)$$

where ρ is the air density ($\frac{\text{kg}}{m^{3}}$), p is the atmospheric pressure (Pa), $M_{a}$ is the molar mass of dry air ($28.96546\times 10^{-3\;}\text{kg}/\text{mol}$), $M_{v}$ is the molar mass of water vapor ($18.01528\times 10^{-3\;}\text{kg}/\text{mol}$), R is the universal gas constant ($8.314472\;\frac{J}{\left(\text{mol}\cdot K\right)}$), T is the absolute temperature in Kelvin (K), $x_{v}$ is the mole fraction of water vapor, Z is compressibility factor, which accounts for the deviation from ideal gas behavior due to the presence of moisture.

We apply a Gaussian convolution to the power curve to account for statistical events in wind speeds, as in Caglayan et al.^63^ RESKit then computes the capacity factor at each turbine location by invoking the convoluted power curve at the given wind speed for each placement location. Based on the capacity factors, we calculate the annual full-load hours for each placement and apply assumed efficiency losses into the results.

Turbine availability refers to the percentage of time that the turbine is operational and able to generate electricity. This includes downtime for maintenance, repairs, grid issues, and other operational constraints. Following research on the relationship between distance from shore and availability in the North Sea,^92^ as well as a recent literature review on large-scale wind resource assessments,^4^ we assume an availability of 92% for the fixed-foundation scenario and an availability of 87% for the mixed-technology scenario. Array efficiency refers to the efficiency of the entire wind park, incorporating electrical losses through the cables and due to turbulence and wake effects. As we assume near-future turbines with a large 10× rotor diameter spacing (to reduce wake losses), we assume the array efficiency as 88%.^30^ Thus, the total loss factor applied to the simulated full load hours is 0.81 for the fixed-foundation scenario and 0.766 for the mixed-technology scenario.

## Resource availability

### Lead contact

Further information and requests for resources and reagents should be directed to and will be fulfilled by the lead contact, Tristan Pelser (t.pelser@fz-juelich.de).

### Materials availability

This study did not generate new unique materials.

### Data and code availability

The software utilized for this study, including all relevant scripts, is hosted on an openly accessible repository on GitHub. The specific scripts used in this study can be found under the example_workflows/offshore_north_sea directory within the repository. The repository can be accessed via the following link: FZJ-IEK3-VSA/ethos.REFLOW (github.com). All necessary data are downloaded when the project scripts are executed. The scripts are also archived on Zenodo.^93^

For further information or to report issues, users are encouraged to visit the GitHub repository or contact the corresponding author.

## Acknowledgments

The authors would like to thank the German Federal Government, the German State Governments, and the Joint Science Conference (GWK) for their funding and support as part of the NFDI4Ing consortium, managed by the 10.13039/501100001659German Research Foundation (DFG) – 442146713. This work was also supported by the 10.13039/501100009318Helmholtz Association as part of the program “Energy System Design.” In addition, we would like to thank the two anonymous reviewers for their comments on an earlier version.

## Author contributions

Conceptualization, T.P., J.M.W., and P.K.; methodology, T.P., J.M.W., and P.K.; formal analysis, T.P.; writing – original draft, T.P.; writing – review & editing, T.P., J.M.W., and P.K.; visualization, T.P.; supervision, J.M.W. and P.K.; project administration, P.K.; funding acquisition, D.S.

## Declaration of interests

The authors declare no competing interests.

## Declaration of generative AI and AI-assisted technologies

During the preparation of this work the authors used the tool “ChatGPT” by OpenAI to check grammar and spelling in a few places, and to make minor improvements to readability and style. After using this tool, the authors reviewed and edited the content as needed, and take full responsibility for the content of the publication.

## Supplemental information

Document S1. Tables S1 and S2

Document S2. Article plus supplemental information
