# Supplementary material for: ETHOS.REFLOW: An open-source workflow for reproducible renewable energy potential assessments
Source: Patterns (N Y). 2025 Feb 4;6(2):101172. doi: 10.1016/j.patter.2025.101172 (PMC11873006; doi:10.1016/j.patter.2025.101172)
Supplement: Document S1. Tables S1 and S2 [file mmc1.pdf]

Patterns, Volume *6*

Supplemental information

ETHOS.REFLOW: An open-source workflow for reproducible renewable energy potential assessments

Tristan Pelser, Jann Michael Weinand, Patrick Kuckertz, and Detlef Stolten

SUPPLEMENTAL INFORMATION

Table S1 National-level installable capacity [GW] and mean annual energy yield (AEY) [TWh] for the fixed-foundation scenario (maximum depth of 50 meters), for both methodologies.

	Installable capacity [GW]		Mean annual energy yield [TWh]	
**Country**	**ERA5, explicit placements**	**NEWA, uniform power density**	**ERA5, explicit placements**	**NEWA, uniform power density**
Belgium	1.94	1.43	7.43	4.59
Denmark	84.45	93.71	348.86	372.63
France	0.66	0.38	2.48	1.35
Germany	48.30	50.12	192.11	180.76
Netherlands	71.18	70.92	277.47	255.34
Norway	3.00	2.22	12.68	7.87
Sweden	0.03	0.03	0.12	0.08
United Kingdom	20.73	18.69	82.12	71.82
Total North Sea	230	238	923	894

Table S2 National-level installable capacity [GW] and mean annual yield (AEY) [TWh] for the mixed-technology scenario (maximum depth of 1000 meters), for both methodologies.

	Installable capacity [GW]		Mean annual energy yield [TWh]	
**Country**	**ERA5, explicit placements**	**NEWA, uniform power density**	**ERA5, explicit placements**	**NEWA, uniform power density**
Belgium	1.94	1.43	7.02	4.34
Denmark	119.60	138.63	469.65	521.29
France	0.66	0.38	2.35	1.28
Germany	48.90	51.20	183.90	174.62
Netherlands	71.72	70.92	264.39	243.43
Norway	216.53	251.16	855.70	843.05
Sweden	6.17	7.39	23.98	23.37
United Kingdom	302.69	340.10	1,154.72	1,236.05
Total North Sea	768	861	2,961	3,047
